# Hepatitis C virus infects and perturbs liver stem cells

**DOI:** 10.1128/mbio.01318-23

**Published:** 2023-11-08

**Authors:** Nathan L. Meyers, Tal Ashuach, Danielle E. Lyons, Mir M. Khalid, Camille R. Simoneau, Ann L. Erickson, Mehdi Bouhaddou, Thong T. Nguyen, G. Renuka Kumar, Taha Y. Taha, Vaishaali Natarajan, Jody L. Baron, Norma Neff, Fabio Zanini, Tokameh Mahmoudi, Stephen R. Quake, Nevan J. Krogan, Stewart Cooper, Todd C. McDevitt, Nir Yosef, Melanie Ott

**Affiliations:** 1 Gladstone Institute of Virology, San Francisco, California, USA; 2 Department of Electrical Engineering and Computer Science and Center for Computational Biology, University of California Berkeley, Berkeley, California, USA; 3 California Pacific Medical Center Research Institute, San Francisco, California, USA; 4 Cellular and Molecular Pharmacology, University of California, San Francisco, California, USA; 5 Gladstone Institute of Data Science and Biotechnology, San Francisco, California, USA; 6 Quantitative Biosciences Institute, University of California, San Francisco, California, USA; 7 Gladstone Institute of Cardiovascular Disease, San Francisco, California, USA; 8 Department of Medicine, University of California San Francisco, San Francisco, California, USA; 9 Chan Zuckerburg Biohub, San Francisco, California, USA; 10 Department of Bioengineering, Stanford University, Stanford, California, USA; 11 Department of Biochemistry, Erasmus University Medical Center, Rotterdam, the Netherlands; 12 Bioengineering & Therapeutic Sciences, University of California San Francisco, San Francisco, California, USA; 13 Ragon Institute of Massachusetts General Hospital, MIT and Harvard, Cambridge, Massachusetts, USA; University of Ljubljana, Ljubljana, Slovenia

**Keywords:** hepatitis c virus, organoid, liver disease, stem cell, single-cell RNA sequencing, chronic infection, hepatocellular carcinoma

## Abstract

Hepatitis C virus (HCV) is the leading cause of death from liver disease. How HCV infection causes lasting liver damage and increases cancer risk remains unclear. Here, we identify bipotent liver stem cells as novel targets for HCV infection, and their erroneous differentiation as the potential cause of impaired liver regeneration and cancer development. We show 3D organoids generated from liver stem cells from actively HCV-infected individuals carry replicating virus and maintain low-grade infection over months. Organoids can be infected with a primary HCV isolate. Virus-inclusive single-cell RNA sequencing uncovered transcriptional reprogramming in HCV^+^ cells supporting hepatocytic differentiation, cancer stem cell development, and viral replication while stem cell proliferation and interferon signaling are disrupted. Our data add a new pathogenesis mechanism—infection of liver stem cells—to the biology of HCV infection that may explain progressive liver damage and enhanced cancer risk through an altered stem cell state.

**IMPORTANCE:**

The hepatitis C virus (HCV) causes liver disease, affecting millions. Even though we have effective antivirals that cure HCV, they cannot stop terminal liver disease. We used an adult stem cell-derived liver organoid system to understand how HCV infection leads to the progression of terminal liver disease. Here, we show that HCV maintains low-grade infections in liver organoids for the first time. HCV infection in liver organoids leads to transcriptional reprogramming causing cancer cell development and altered immune response. Our finding shows how HCV infection in liver organoids mimics HCV infection and patient pathogenesis. These results reveal that HCV infection in liver organoids contributes to liver disease progression.

## INTRODUCTION

Hepatitis C virus (HCV) is a leading cause of hepatocellular carcinoma (HCC) (1). Approximately 71 million individuals are chronically infected with HCV and at risk of liver disease, including fibrosis, steatosis, cirrhosis, and HCC (2). Direct-acting antivirals (DAAs) effectively eradicate HCV (3, 4), but they do not reverse terminal liver disease, and treated individuals remain at risk for HCC (4, 5). In the healthy liver, resident bipotent adult stem cells regenerate the tissue after injury by giving rise to hepatocytes and ductal cells (6–8). The persistence of liver disease after virus eradication may therefore indicate that these stem cells have sustained durable damage that prevents them from regenerating the tissue lost to infection. Indeed, in chronically infected HCV patients, chronic intrahepatic inflammation activates the proliferation of liver stem cells (9), which is linked to the development and progression of fibrosis (10) and is at the origin of primary liver cancers (7). These findings suggest that liver stem cells are altered by infection, in ways that could lead to lasting liver damage in patients with chronic HCV infection even after treatment with DAAs. But whether durable alterations of the liver stem cells result from chronic liver inflammation or also from direct infection remains unclear.

Demonstrating that HCV can infect liver stem cells is challenging. HCV and especially primary viral isolates are difficult to culture *ex vivo*. Long-term replication of HCV in adult or fetal primary human liver cells is hampered by the progressive de-differentiation of liver cells in culture and a robust interferon response in primary human liver cells preventing viral replication (11–16). Many studies use cell culture-adapted strains in a Huh 7-derived hepatoma cell line with defective interferon signaling. However, these cells lack proper cell polarity and display abnormal lipid metabolism (17–20). Hepatocyte-like cells from human-induced pluripotent stem cells (iPSCs) offer a more physiological model (4, 14, 15, 21, 22). However, iPSCs are refractory to HCV infection due to high intrinsic expression of interferon-stimulated genes (ISGs). The same problem arises with fetal hepatic progenitor cells (14–16).

To overcome these barriers, we examined adult liver stem cells of individuals infected with HCV. Stem cells with bipotent characteristics analogous to those of hepatic progenitor cells can be cultured *ex vivo* in a 3D organoid form (23, 24). 3D liver organoids maintain bipotency, with the ability to differentiate into hepatocyte- and cholangiocyte-like cells, and cell polarity and reproduce *in vitro* the *in vivo* phenotype of several liver diseases (23–25). We therefore generated bipotent liver stem cell organoids from HCV-infected individuals. We found that the organoids carried HCV and successfully maintained HCV infection in long-term culture, opening the intriguing possibility that direct infection of liver stem cells contributes to the hepatopathogenesis of HCV infection.

## RESULTS

### Successful generation of liver stem cell organoids from HCV-infected individuals

In order to determine if adult liver stem cell organoids could be derived from HCV^+^ donors, we generated organoids from liver resections from six donors (Table 1) as described (23, 24). Three donors had no viral infection (NV) but various forms of metastatic cancers. Three donors were viremic, presented with different HCV genotypes (HCV1–3), and had virus-associated HCC. Donors were 63–70 years old with one female and two males in each group.

**TABLE 1:** Liver organoids grow from non-viral and HCV-infected individuals^*a*^

Culture name	Donor phenotype	HCV^+/−^ at resection
NV1	70 yo male, colonic adenocarcinoma, minimal fibrosis	−
NV2	68 yo male, alcoholic-cholangiocarcinoma, bridging stage 3 fibrosis	−
NV3	66 yo female, angiosarcoma, no fibrosis	−
HCV1	68 yo male, HCV GT 1, two times treatment failure (interferon and Harvoni), HCC, advanced fibrosis	+
HCV2	66 yo female, HCV GT 2b, HCC, cirrhotic	+
HCV3	63 yo male, HCV GT 3, HCC, cirrhotic	+

^*a*^ Liver organoids generated from six donors, using single-cell digests from patient liver resections. Three non-viral (NV) patients with various metastatic cancers that necessitated liver resection. Three donors were HCV patients that were viremic at the time of resection. Their HCV genotypes were known and match donor number (i.e., HCV1, 2, 3). yo, years old.

**Fig 1:**
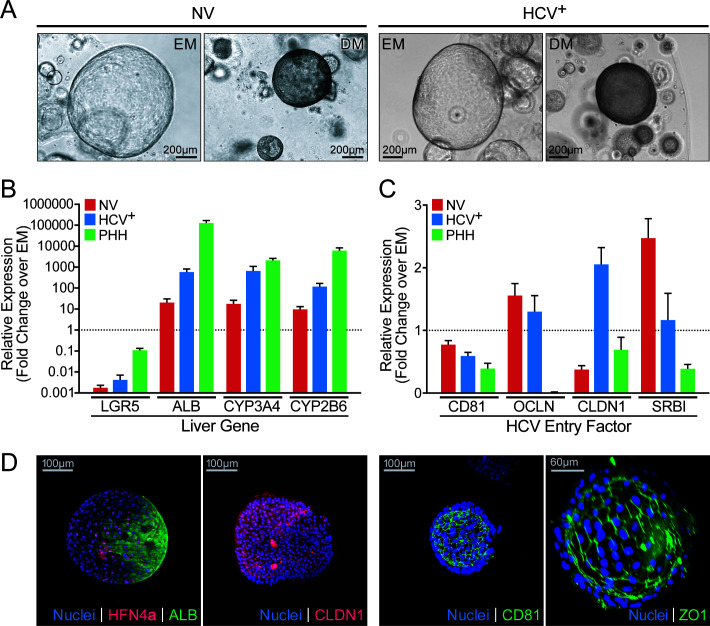
Liver organoids grow from HCV-infected individuals and show similar differentiation potential. (**A**) Representative brightfield microscopy images of liver organoids grown from uninfected (NV) or HCV^+^ donors are shown in the stem cell (EM) and differentiated (DM) states. Organoids are morphologically distinct in EM vs DM states, but each state was morphologically identical across all six NV and HCV^+^ donors. (**B**) Quantitative PCR (qPCR) quantification of hepatocyte stem cell marker LGR5 and hepatocyte markers ALB, CYP3A4, and CYP2B6 in DM organoids from three NV donors and three HCV^+^ donors, and in two primary hepatocyte samples relative to EM. For each gene, data were pooled from n ≥ 2 biological replicates per organoid or hepatocyte donor and represented as mean ± SD. Transcript expression was normalized to 18S and plotted as a fold change over the gene’s expression in EM (ΔΔC_T_) which was set to one and is marked by a dotted line. Fold change was plotted on a log10 scale. (**C**) qPCR quantification of HCV entry markers, CD81, OCLN, CLDN1, and SR-B1 in the same samples as in (**B**). EM expression levels were set to one (marked by a dotted line), and fold change in DM was plotted on a linear scale. (**D**) Representative light-sheet microscopy images are shown for differentiated liver organoids (DM) stained for hepatocyte markers HNF4α and ALB, HCV entry factors CLDN1 and CD81, or apical membrane marker ZO1.

NV and HCV^+^ organoids grew in a progenitor state in expansion medium (EM) with a similar lifespan of ~4 months in culture (Fig. 1A; Fig. S1). They showed the typical hollow structure with an epithelial monolayer bounding a cell free inside (Fig. 1A) and differentiated toward a hepatocyte-like fate when switched to differentiation medium (DM) (where Wnt and Notch agonists were replaced with Notch inhibitor DAPT, dexamethasone, and transforming growth factor β [TGFβ] and bone morphogenic protein [BMP] agonists [24, 25]). Upon differentiation, proliferation slowed as expected and organoids became opaque and transitioned from an epithelial monolayer to a pseudo-stratified epithelium (24, 25). No phenotypic differences were observed between NV and HCV^+^ organoids regardless of differentiation status (Fig. 1A and D). Differentiation of both NV and HCV^+^ organoids showed similar induction of mRNA expression of several hepatocyte markers, albumin (ALB) and cytochrome P450 enzymes (CYP3A4 and CYP2B6), although to a lesser extent than primary human hepatocytes from noninfected donors, and decreased expression of the stem cell marker LGR5 (Fig. 1B). Transcripts for HCV entry factors including cluster of differentiation 81 (CD81), occludin (OCLDN), claudin-1 (CLDN1), and scavenger receptor class B type I (SR-B1) were more highly expressed in organoids than in primary human hepatocytes with no consistent differences associated with infection status and at similar levels before and after differentiation (Fig. 1C). CD81 was properly expressed and located at tight junctions and the apical membrane (Fig. 1D). Differentiated organoids from both groups expressed albumin and the hepatocyte nuclear factor 4⍺ (HNF4A) proteins, the HCV entry factor claudin-1, and were polarized with the apical membrane, marked by zonula occludens-1 (ZO-1), facing the organoid lumen (Fig. 1D). These results demonstrate that liver organoids can be generated from HCV^+^ donors, and they display similar organoid-forming properties as those derived from HCV^–^ donors.

### Single-cell transcriptional profiling of organoids from HCV-infected donors

**Fig 2:**
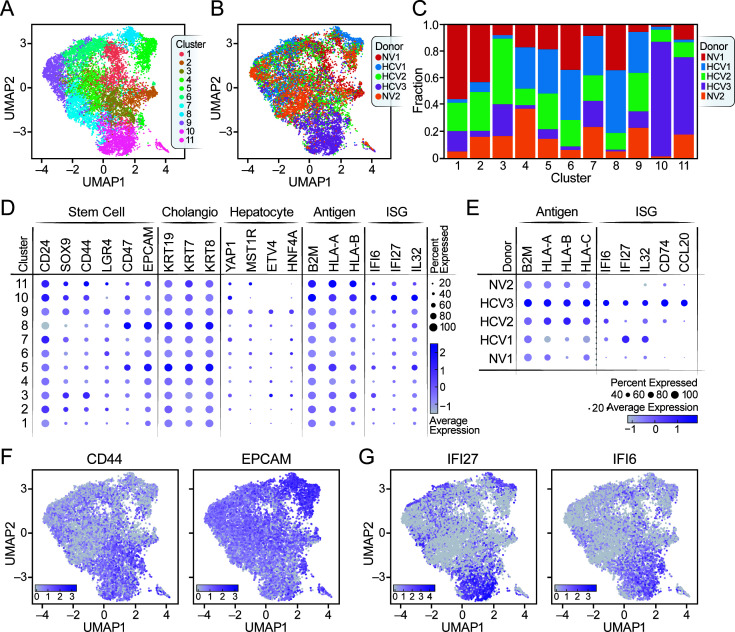
Undifferentiated EM liver organoids derived from HCV^+^ donors up-regulate antigen presentation and interferon-stimulated genes. (**A and B**) Uniform manifold approximation and projection (UMAPs) of 3′ scRNA-seq of EM organoids from five donors are plotted with cells labeled by cluster (**A**) and donor (**B**). (**C**) A stacked bar plot shows the donor composition of each cluster. Clusters are shown on the x-axis and the fraction of cells from each donor within that cluster is plotted on the y-axis. Donor color matches the colors used in panel **A**. (**D**) A dot plot shows expression (as pseudolog) by cluster of cancer stem cell markers (stem), ductal, hepatocyte, and antiviral markers, including antigen presentation genes (antigen) and interferon-stimulated genes (ISGs). (**E**) A dot plot shows gene expression by donor (as pseudolog) for antigen presentation genes and ISGs. (**F and G**) UMAPs for gene expression (as pseudolog) of two of the stem cell markers (CD44 and EpCAM) (**F**) and two of the ISGs (IFI27 and IFI6) (**G**). These plots superimpose the UMAP plot in (**A**) with the relative expression levels of a given gene.

To further search for possible differences between NV and HCV^+^ organoids, we performed single-cell RNA sequencing (scRNA-seq) on EM organoids from two NV and three HCV^+^ donors using the 10X Genomics Drop-Seq protocol (Fig. 2; Data S1) (26). We analyzed the combined data sets (Table S1) using single-cell variational inference (scVI), a deep generative model for analyzing scRNA-seq data, which accounts for limited sensitivity and batch effects (27), followed by clustering and annotation using Vision (28). We identified 11 clusters containing cells from the five organoid cultures. Cells from different donors were well mixed across clusters without any specific clustering based on infection status of the donor, except for cells from the HCV3 donor, which were less well distributed and mostly enriched in clusters 10 and 11 (Fig. 2). Of note, HCV RNA is not poly-adenylated, and hence not captured by the standard poly-T capture oligonucleotide in the 10X Genomics Drop-Seq protocol.

Since EM organoids maintain a stem cell-like state, we focused on expression of stem cell markers (Fig. 2). While CD24 was expressed in most clusters, other stem cell markers were enriched in distinct subsets: SOX9, CD44, and LGR4 expression marked clusters 3, 9, 10, and 11, whereas CD47 and EpCAM were highly expressed in clusters 5 and 8. The latter clusters were enriched for keratin (KRT) 19, KRT7, and KRT8, indicating a cholangiocytic phenotype (29, 30). Cluster 9 expressed YAP1, MST1R, ETV4, and HNF4A, all associated with a hepatocytic fate (Fig. 2C) (29). These results demonstrate the bipotent nature of EM organoids, with distinct stem cell populations primed toward either a cholangiocytic or a hepatocytic fate (23, 24). Notably, this underscores the relatively simple cellular architecture of liver organoids in contrast to other more complex organoid systems such as adult stem cell-derived gut organoids which contain several different cell types: paneth cells, goblet cells, M-cells, enterocytes, and tuft cells (31, 32).

When gene expression was analyzed by organoid donor, antigen presentation genes and ISGs were higher in HCV^+^ than NV organoids, with ISGs specifically up-regulated in organoids from HCV1 and HCV3 donors. ISG expression was not observed in NV organoids but was successfully induced after interferon or treatment (Fig. S2). Thus, the interferon response is intact in organoids derived from adult liver stem cells, and its constitutive activation in HCV1- and HCV3-infected organoids indicates specific innate immune activation and possible viral infection in these organoids.

### Stem cell organoids can carry low-grade replicating HCV

**Fig 3:**
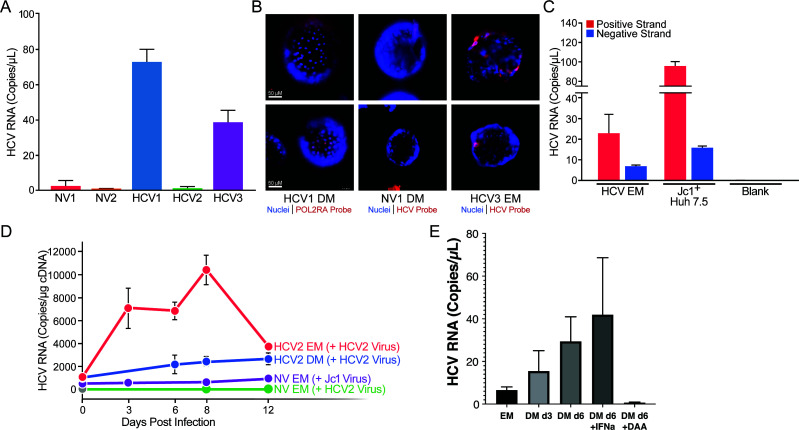
HCV infection persists in EM organoids from viremic donors. (**A**) ddPCR quantification of HCV RNA in EM organoids using a primer and probe set unique to the HCV 5´ untranslated region (UTR). Data are from n ≥ 3 biological replicates per organoid donor and represented as mean ± SEM. (**B**) RNAScope of organoids using POL2RA or HCV RNA probes (red). Nuclei were stained with Hoescht (blue). (**C**) ddPCR quantification of positive and negative HCV strands in HCV1 organoids and HCV-infected Huh 7.5 cells. Data are from n ≥ 3 biological replicates per organoid donor and represented as mean ± SEM. (**D**) qPCR quantification of HCV RNA from EM and DM HCV2 organoids infected with autologous virus from the HCV2 donor and from NV EM organoids infected with virus from HCV2 or Jc1, over 12 days. EM organoids were passaged at day 8, resulting in a lower HCV concentration at day 12. In parallel, EM organoids from non-viral donors were spin-infected with the HCV2 viral isolate at the same multiplicity of infection (MOI) and with a JC1 lab strain at MOI = 5. Data are represented as mean ± SD. (**E**) Differentiation of organoids re-stimulates viral production. HCV organoids at >2 months in culture were differentiated for 6 days in the presence or absence of interferon α (IFN-α) (1,000 U/mL) or a combination of the direct-acting antiviral (DAA) drugs sofosbuvir (10 µM) and velpatasvir (100 nM). Intracellular RNA was collected and analyzed by ddPCR for HCV. Results are pooled from *n* = 3 experimental replicates.

To test for a possible low-grade viral infection in the HCV^+^ organoids, we developed a droplet digital real time (RT-PCR assay to sensitively measure HCV RNA levels in cultures (Fig. 3A). Briefly, cDNA from organoids was mixed with Droplet Digital PCR (ddPCR) supermix and primers specific to HCV for droplet generation by the QX100 droplet generator, thermocycler amplification, and detection by the QX100 droplet reader. For detection of the negative strand, a forward primer with a unique tag was used to generate cDNA that was used for ddPCR with primers selective for that tag. Using this method, HCV RNA was detected in HCV1 and HCV3, but not in NV or HCV2 organoids, at a copy number of ~40–80 copies per microliter. For comparison, cultures infected with the lab-adapted Jc1 strain of HCV produce ~100 copies per microliter (Fig. 3C). RNAScope analysis using an RNA probe against HCV successfully detected HCV RNA in approximately 20% of cells for both HCV1 and HCV3 organoids (Fig. 3B). To ensure we detected active replication, we adapted the digital droplet PCR protocol to the negative strand of HCV RNA, an intermediate of viral replication (33). HCV negative-strand RNA was detected in infected HCV1 and HCV3 EM organoids as well as in replication-competent Huh 7.5 hepatoma cells infected with the HCV-Jc1 clone, confirming active RNA replication in these two samples of EM organoids (Fig. 3C). Infection was observed over months in EM cultures of HCV1 but declined progressively to undetectable levels before the natural endpoint of the organoid life span (~6 months) (Fig. S1A and S2C). Interestingly, virus was not lost when RNA levels were undetectable because when EM cultures were differentiated with DM media at this point, viral RNA levels recovered, a process successfully suppressed with a complex combination of DAAs (sofosbuvir and velpatasvir), but not interferon or treatment (Fig. 3E). Notably, the HCV1 donor underwent several unsuccessful interferon treatment cycles and reacted only to a combination treatment of sofosbuvir and velpatasvir. Collectively, these results confirm that ISG production in HCV1 and HCV3 organoids is accompanied by active infection, which persists even when RNA can no longer be detected with available methods, pointing to a possible reservoir function of liver stem cells.

Next, we focused on the HCV2 organoids, where no infection was detected (Fig. 3A). Notably, cells from HCV2 did not show any phenotypic, molecular, or gene expression differences that separated them from others in Fig. 1 and 2. As we had access to banked serum samples for the HCV2 donor, we utilized those to inoculate HCV2 EM and DM cultures by spin infection (Fig. 3D). In both cultures, viral RNA was detected by quantitative RT-PCR over 2 weeks; stem cell cultures (EM) cultures were more robustly infected than more differentiated (DM) cultures, supporting the model that stem cells are bona fide infection targets for HCV. Notably, HCV2 organoid cultures were not infectable with the lab-adapted Jc1 strain or with serum from the HCV1 donor, highlighting a possible virus:host adaptation that may restrict infectivity in primary cell culture. This was further underscored by the fact that the HCV2 inoculum that successfully infected HCV2 organoids did not infect EM organoids from NV donors (Fig. 3D). These data show that liver stem cells from HCV^+^ donors are permissive to *ex vivo* infection with an autologous viral isolate.

### HCV infection drives clustering in virus-inclusive scRNA-seq

**Fig 4:**
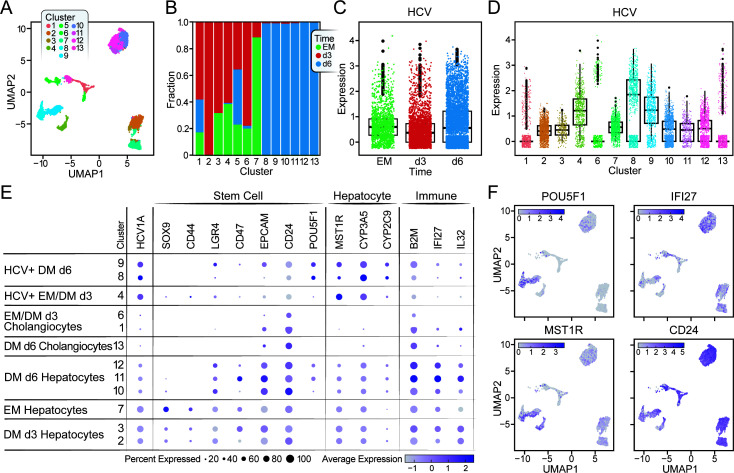
HCV infection of liver stem cells enhances differentiation to a hepatocyte-like fate. (**A**) A UMAP of HCV1 EM, day 3 (d3) DM, and day 6 (d6) DM organoids is generated from the 5´ scRNA-seq data with HCV capture oligo and plotted with cells identified by the 13 clusters from scVI analysis. Cluster 5 is omitted due to low read quality. (**B**) A stacked bar plot (as pseudolog) shows the composition by sample of each of the 13 clusters identified in scVI. Clusters are shown on the x-axis and the fraction of cells from each donor within that cluster is plotted on the y-axis. (**C**) A box plot shows HCV RNA expression on the y-axis (as pseudolog) across the three HCV1 samples on the x-axis. (**D**) The same UMAP in (**A**) is plotted with cells identified by the 13 clusters from scVI analysis. Cluster 5 is omitted due to low read quality. (**C**) HCV RNA expression is plotted (as pseudolog) on the UMAP. (**E**) Dot plot shows gene expression (as pseudolog) of HCV (Viral), stem cell (Stem), hepatocyte, and immune markers. Clusters are on the y-axis, and are further labeled with identities assigned from gene expression analysis. (**F**) UMAPs are shown for gene expression (as pseudolog) of POU5F1, CD24, MST1R, and IFI27.

Since we find only ~20% of cells infected in the organoids and to distinguish the transcriptional profile of infected vs uninfected cells, we designed a viral capture assay using a synthetic oligonucleotide corresponding to a conserved region of the HCV sequence in the Chromium Single-Cell 5´ Gel Bead & Library Kit (17–19). The protocol was established and worked in Huh 7.5 cells infected with HCV-Jc1 (Fig. S3). Next, we applied viral capture scRNA-seq to HCV1 EM cells under standard EM culture conditions or undergoing differentiation for 3 or 6 days. scVI analysis of the combined data sets identified 13 clusters (Fig. 4A and B; Data S2). Average HCV RNA expression was similar across the three differentiation stages, with an average of fewer than 10 copies per cell (Fig. 4). Cells with the highest HCV load (HCV^high^ cells) were in just three of the 13 clusters (Fig. 4A through D): cluster 4, which comprised infected EM and day 3 DM cells; and clusters 8 and 9, which comprised infected day 6 DM cells. These data support a model where HCV infection alters gene expression in liver stem cells at their basal state and during differentiation.

### Differentiation of uninfected cells shows divergence between hepatocytic and cholangiocytic lineages

Uninfected or HCV^low^ cholangiocytic and hepatocytic progenitor cells were enriched in clusters 7, 2, and 3. Within these clusters, EM cells (cluster 7) highly expressed hepatocyte progenitor markers, such as SOX9 and HNF4A (34), TACSTD2 (TROP2), a gene expressed in bipotent liver progenitor cells, and CXCL8, a gene expressed in progenitor cells primed toward cholangiocyte differentiation (35) (Fig. 4E and F; Fig. S4A and B). On day 3 of differentiation, SOX9, CD44, TACSTD2, and CXCL8 were down-regulated (clusters 2 and 3), but hepatocyte markers, such as GLUL and HAMP, were not yet up-regulated (30, 35) (Fig. 4E; Fig. S4A). Up-regulation of GLUL and HAMP occurred in uninfected day 6 DM cells (clusters 10–13), indicating these clusters include the most mature hepatocyte-like cells (Fig S4A).

After differentiation, cholangiocyte-like cells had low expression of stem cell and hepatocyte markers but high expression of cholangiocyte markers, such as KRT8/19 and clustered in clusters 1 and 6 and part of cluster 13 (Fig. 4E; Fig. S4A). Notably, these clusters showed the lowest HCV infection rate, indicating that the cholangiocytic state may not support HCV infection (Fig. 4C, D, and E).

### HCV infection primes liver stem cells toward the hepatocytic fate

We next compared the differentiation status of HCV^high^ clusters (4, 8, and 9) to that of lowly infected clusters. HCV^high^ clusters showed low expression of hepatic stem cell markers, such as SOX9, CD44, LGR4, CD47, EpCAM, and CD24, at all stages of differentiation, including in the EM state (Fig. 4E). In contrast, hepatocyte markers such as MST1R, CYP3A5, and CYP2C9 were strongly expressed in all HCV^high^ clusters (Fig. 4E and F). These results suggest that HCV may perturb the stemness of progenitor cells to preferentially prime them toward a hepatocyte fate.

To exclude the possibility that clusters 4, 8, and 9 represent a subset of cells that occur during normal differentiation and are more permissive to HCV infection, we examined differentiated organoids from NV donors. scRNA-seq analysis of two NV organoids identified 14 unique clusters of cells (Fig. S5A). However, none showed an expression pattern matching that of HCV^high^ clusters (Fig. S5C), supporting the model that HCV infection, indeed, perturbs stem cell differentiation.

### Up-regulation of cancer stem cell marker OCT4 in infected cells during differentiation

One notable difference between HCV^high^ and HCV^low^ clusters was that upon differentiation, HCV^high^ cells in clusters 8 and 9 up-regulated expression of POU5F1 (octamer-binding transcription factor 4 [OCT4]) (Fig. 4F), a master regulatory transcription factor defining embryonic stem cells and usually absent from liver progenitor cells or lowly expressed during differentiation (36). When up-regulated in tissue progenitor cells, OCT4 promotes a cancer stem cell state (37). These data support a model where HCV infection of liver stem cells dysregulates differentiation into hepatocyte-like cells with a possible cancer stem cell identity.

### Blunted interferon response in HCV-infected cells with and without differentiation

We further compared expression of interferon-stimulated and antigen presentation genes between HCV^low^ and HCV^high^ clusters, including IFI27, IFI6, and B2M (Fig. 4E and F). In stem cells, expression of all three genes was lower in HCV^high^ compared to HCV^low^ clusters. Expression increased during differentiation of HCV^low^ cells and was highest in cluster 11 comprised of mostly uninfected DM d6 hepatocytes (Fig. 4E). However, in HCV^high^ clusters, expression did not increase on differentiation. These results indicate that expression of interferon-stimulated and antigen presentation genes is blunted in HCV-infected cells compared to uninfected bystander cells at all stages of differentiation. They also underscore results from previous studies reporting increased interferon responses upon cellular differentiation (16).

### Network propagation identifies alterations in splicing, ATP synthesis, and ribosome biogenesis pathways in HCV^high^ clusters

**Fig 5:**
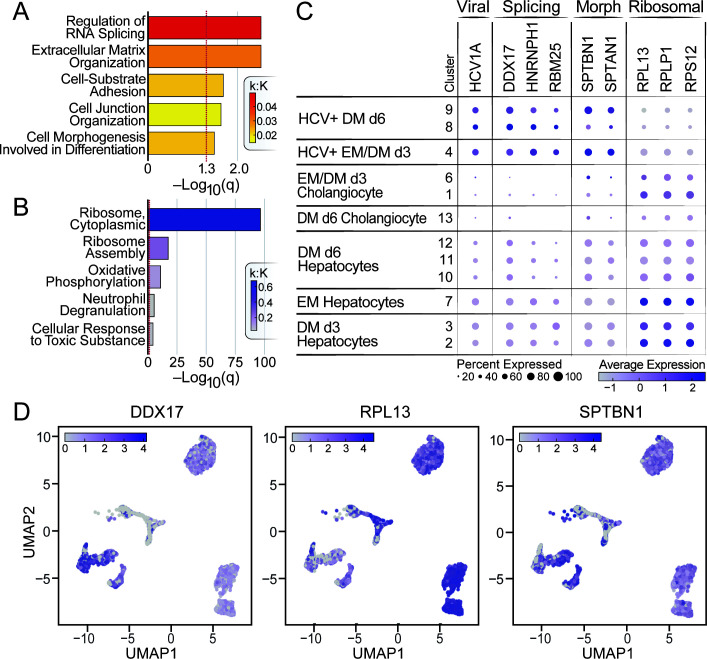
HCV infection of liver stem cells up-regulates pro-viral factors and RNA splicing genes. Metascape gene ontology term enrichment analysis identifies pathways from the 81 genes commonly up-regulated (**A**) or down-regulated (**B**) in at least two of the HCV^high^ (4, 8, and 9) clusters. Terms are ordered by q-value and colored based on the observed genes within the term (K) divided by the total genes in the term (K). (**C**) A dot plot is shown for genes in different gene ontology (GO) terms: RNA splicing (splicing), cell morphogenesis involved in differentiation (morph), and ribosome. Clusters on the y-axis and labeled as in Fig. 4. (**D**) UMAPs show expression (as pseudolog) of three of the top dysregulated genes: DDX17, RPL13, and SPTBN1.

To identify the main transcriptional targets of HCV infection, we compared the top 150 up- or down-regulated genes from each HCV^high^ cluster. We found 81 that were up-regulated and 142 down-regulated within at least two clusters, relative to uninfected clusters (Fig. S6A and B). Based on gene ontology term enrichment analysis, up-regulated pathways included RNA splicing, extracellular matrix organization, and cell morphogenesis involved in differentiation (Fig. 5A; Fig. S6), and down-regulated pathways included ribosomal genes and oxidative phosphorylation (Fig. 5B; Fig. S6) (38).

**Fig 6:**
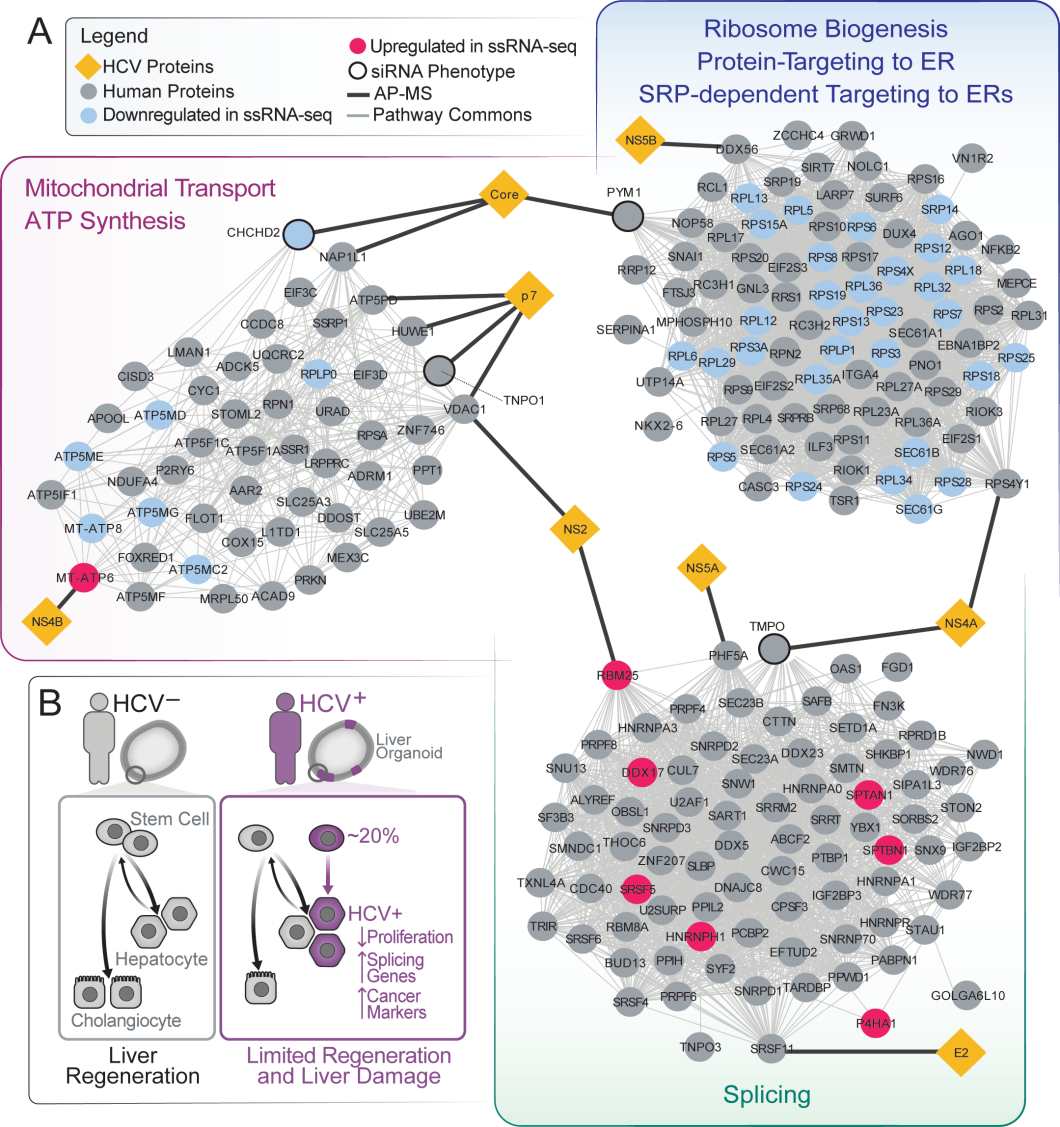
Genes regulated by HCV infection of liver stem cells form networks with HCV interacting proteins. (**A**) Network analysis of commonly up- or down-regulated genes from 5A and 5B and previously published HCV interactome. (**B**) Model of HCV infection in liver stem cells.

We noted that many up- or down-regulated genes were also detected in a previously published HCV protein interactome in Huh 7.5 cells (39). In particular, a network propagation analysis between the commonly differentially expressed genes in the scRNA-seq and the HCV protein interaction partners revealed three major converging molecular networks: ribosome biogenesis and mitochondrial transport/ATP synthesis, comprised of mostly down-regulated genes, and splicing, composed of mostly up-regulated genes (Fig. 6A). This observation indicates that viral infection intersects these cellular pathways at multiple steps.

Down-regulation of pathways involved in ribosome biogenesis, mitochondrial transport/ATP synthesis, and oxidative phosphorylation suggests that HCV^high^ cells have reduced proliferation rates (40) (Fig. 5 and 6; Data S3). Up-regulated pathways such as splicing and cell morphogenesis support the notion that HCV positive cells have altered differentiation (41–46).

In summary, transcriptional reprogramming is observed in HCV-infected organoids, which skews differentiation, reduces proliferation, antagonizes interferon signaling, promotes tumorigenesis, and supports viral replication.

## DISCUSSION

Stem cells have so far not been considered a significant reservoir for HCV infection in the liver given reports of high constitutive levels of ISG expression in iPSCs and the difficulty in obtaining culturable material (11, 13). By generating adult stem cell-derived organoids from donors that were actively replicating HCV at the time of liver resection and keeping these organoids for months in culture, we demonstrate that liver stem cells represent novel, long-lived HCV-producing cells *ex vivo* and possibly *in vivo*. Notably, we showed that the three predominant genotypes of HCV, HCV 1, 2, and 3, can each replicate in liver stem cells as we examined donors infected with the three different genotypes.

HCV2 organoids did not carry the virus but were infectable *ex vivo* with the autologous viral isolate obtained from the same donor. We did not find any cellular or molecular characteristics that differentiated HCV2 from HCV1 and 3 as shown by microscopy, RT-qPCR and scRNA-seq. A possibility is that HCV2 does not or to a lesser extent infect liver stem cells *in vivo,* and the virus was therefore not transmitted to the resulting organoids. Consistent with this hypothesis, individuals infected with HCV2 respond usually well to treatment and compared to HCV1b- and HCV3-infected individuals carry a lesser risk for hepatocellular carcinoma (47–49). However, HCV2 organoids in the stem cell state or after differentiation were readily infectable with HCV2 *ex vivo,* excluding liver stem cells that are generally resistant to HCV2 viral infection. The fact that HCV2 only replicated in stem cell organoids from individuals infected with the same virus and not from uninfected donors was surprising and warrants further investigation. It indicates that during chronic infection, HCV may uniquely adapt to best replicate in its host, which may explain why the HCV2 isolate successfully infected HCV2, but not NV organoids. By performing virus-inclusive scRNA-seq of infected and differentiating stem cell organoids, we find characteristic differences between HCV^+^ and HCV^−^ cells.

1. HCV^high^ clusters lack ISG expression, consistent with the notion that HCV infection effectively antagonizes interferon signaling, i.e., through its core and NS3/4 proteins (50). However, HCV modulates the interferon response only in infected, but not in uninfected, stem cells and hepatocyte-like cells, which may limit viral spread to bystander cells with active interferon responses. Overall, adult stem cell-derived organoids showed intact interferon responses to exogenous interferon treatment and viral infection, albeit lower in EM cultures than in DM cultures, possibly explaining increased infection by HCV in stem cell organoids. Constitutive high ISG expression was not observed in adult stem cell-derived organoids. Interestingly, EM organoids harboring HCV1 and HCV3 showed different patterns of ISG and major histocompatibility complex (MHC) gene expression. These differences could reflect the differing viral genotypes or donor genetic backgrounds, which will both be further explored using the organoid platform.
2. Aside from silencing ISGs, HCV infection of stem cells perturbs the expression of differentiation genes. For instance, HCV^high^ clusters up-regulate hepatocyte markers including CYP2C9, which was previously shown to be higher in HCV^+^ versus HCV^–^ non-tumoral liver tissue (51). Further, HCV^high^ clusters up-regulate several genes that influence cell differentiation including SPTNB1, a gene which negatively regulates proliferation of quiescent hepatocytes after partial hepatectomy (52, 53), and numerous genes for RNA splicing. Notably, alteration of splicing in adult mouse livers led to impaired hepatocyte maturation (42), supporting a model where HCV infection perturbs proper liver regeneration by manipulating RNA splicing. Splicing factors DDX5, DDX17, and HNRNPH1 facilitate cell fate determination (44–46) and prevent the epithelial-mesenchymal transition when expressed at high levels (46). RNA splicing proteins also interact with several HCV proteins. During HCV infection, DDX5 and HNRNPH1 are translocated from the nucleus to the cytoplasm by HCV NS5B or core proteins to promote viral replication (54–56). Our results suggest that HCV infection up-regulates these factors to promote its replication while also skewing differentiation toward a hepatocytic fate.
3. At the same time, HCV^high^ cells strongly down-regulate stem cell markers except for POU5F1 (OCT4). HCV core protein regulates expression of OCT4 in HCV^+^ HCC cells, and increased OCT4 expression drives cell-cycle progression (57). The down-regulation of normal liver stem cell factors with up-regulated OCT4 may allow HCV-infected stem cells to replicate without maintaining the normal regenerative properties of hepatocytes, which would facilitate the development of cancer stem cells. Future studies are necessary to explore the transcriptome as well as OCT4 expression of infected hepatocyte-like cells after viral clearance as shown with HCV1 organoids treated with DAAs. This will clarify if these changes persist beyond active replication and could explain why the cancer risk of infected individuals after viral clearance remains elevated (58). Alternatively, we show that very low levels of virus can persist in liver stem cell organoids even below the threshold of detection, which can be reactivated during differentiation. A possible reservoir function of liver stem cells *in vivo* requires further investigation.

Combined, our observations imply that HCV^+^ hepatocyte-like cells have reduced proliferative and trans-differentiation capacities, which could hamper their ability to regenerate liver tissue and increase the potential for liver damage in chronically infected patients. Our gene expression findings suggest a model in which infection with HCV alters the differentiation of bi-potent liver stem cells by dampening cellular proliferation and mitochondrial function while up-regulating cellular splicing, hepatocyte markers and pluripotent stem cell factor OCT4 (Fig. 6B).

Our study is limited by small sample size and low viral replication in the organoids. Since most HCV patients are now treated with direct-acting antivirals, which usually eradicate the virus, access to liver tissue from donors carrying active HCV is limited. Moreover, HCV replicates at approximately 50- to 100-fold lower levels in liver organoids as compared to transformed Huh 7.5 hepatoma cells. Therefore, ultrasensitive assays including ddPCR, RNA-Scope, and virus-inclusive scRNA-seq are necessary to track HCV replication in organoids, but only allow limited studies of the viral life cycle. Future studies will explore whether stem cell reprogramming is dependent on active viral replication or may persist beyond viral clearance, which would explain lasting liver damage and altered cancer risk in individuals freed of the virus.

## MATERIAL AND METHODS

### Culture of cell lines

293T-HA-R-Spondin1-Fc cells were purchased from Trevigen (catalog number 3710–001-K) and cultured according to the manufacturer’s protocol to generate conditioned medium of R-spondin-1. Briefly, cells were grown in selection growth medium (DMEM with 10% fetal bovine serum [FBS], 1% penicillin-streptomycin, 1% glutamine, and 100 mg/mL Zeocin) for >5 days until they were >90% confluent. Medium was replaced with organoid basal medium (Advanced DMEM/F12 from Invitrogen supplemented with 1% penicillin-streptomycin, 1% Glutamax, and 10 mM HEPES). After 3 days, cell supernatant (i.e., R-spondin-1 conditioned medium) was collected, centrifuged at 3,000 × *g* for 15 min, filtered through a 0.22 µm filter, and frozen at −20°C in 10 mL aliquots. This process was repeated by adding fresh organoid basal medium to the cells and collecting supernatant after 4 days.

Huh 7.5 cells were provided by Charles M. Rice and grown under standard conditions.

### Culture of human liver organoids

Liver organoids were generated from bipotent liver stem cells as described (23, 24). Briefly, single cells were isolated from the healthy resection margins of liver samples obtained during partial hepatectomy. After tissue digest, the heterogeneous mixture of single cells was either directly plated or further enriched for EpCAM+ cells using the EasySep Human EpCAM Positive Selection Kit and then plated. For plating, cells were suspended in cold organoid basal medium and mixed with two parts reduced growth factor BME2 (Basement Membrane Extract, Type 2, Trevigen, catalog number 3533–001-02). From this mixture, 50 µL drops containing 1,000–20,000 cells were seeded in 24-well suspension culture plates (Greiner Bio-One, catalog number 662–102). Drops were incubated at 37°C for >20 min and solidified. After this, 500 µL of expansion medium was added to each well. EM is organoid basal medium supplemented with 1% N_2_ and 1% B27 (both from Gibco), 1 mM N-acetylcysteine (Millipore Sigma), 10 nM [leu^15^]-gastrin I human (Millipore Sigma), 10% (vol/vol) R-spondin1 conditioned medium, and 10 mM nicotinamide (Millipore Sigma). EM additionally contains 50 ng/mL recombinant human epidermal growth factor (EGF), 25 ng/mL recombinant human hepatocyte growth factor (HGF), 100 ng/mL recombinant human fibroblast growth factor 10 (FGF10), 10µM forskolin, and 5µM A83-01 (all from Stem Cell Technologies). EM was replaced every 3–4 days.

After 14–21 days, organoids were passaged. For this, cold basal medium was used to collect organoids in 15 mL Falcon tubes and dissolve BME2. Tubes were centrifuged at 250 × *g* for 5 min at 4°C. Medium was aspirated, TrypLE (Gibco) was added to the organoids, and the mixture was incubated at 37°C for 5–10 min. Organoids were further dissociated by pipette mixing and then diluted in cold basal medium. After another spin and medium aspiration, cells were mixed with BME2 and seeded into new drops. After this initial passage, organoids were passaged every 2 weeks. Stocks of early-passage (P1 to P3) organoid lines were prepared by dissociating organoids, mixing them with recovery cell culture freezing medium (Gibco), and frozen, following standard procedures. These samples could be thawed and immediately cultured, using EM supplemented with 10 µM Y-27632 (Stem Cell Technologies) for the first 3 days after thawing.

To induce differentiation to a hepatocyte-like fate, EM was supplemented with 25 ng/mL BMP7 (ProSpec) for 3–4 days and then changed to differentiation medium. DM is basal medium supplemented with 1% N_2_, 1% B27, 1 mM N-acetylcysteine, 10 nM [leu^15^]-gastrin I human, 50 ng/mL EGF, 25 ng/mL HGF, 0.5 µM A83-01, 25 ng/mL BMP7, 10 µM DAPT (Stem Cell Technologies), 3 µM dexamethasone (Millipore Sigma), and 100 ng/mL recombinant human FGF19 (ProSpec). DM was changed every 3–4 days for a period of 3–15 days.

### Real-time quantitative PCR

RNeasy kits from Qiagen were used for RNA extraction and isolation. To extract RNA, medium was aspirated from the well to leave organoids suspended in BME2. Three hundred fifty microliters of buffer RLT (lysis buffer) was added directly to the well. After a short incubation for RLT to dissolve BME2, sample lysate was transferred to a 1.5 mL Eppendorf tube. Lysate was either stored at −80°C for up to 1 month or extracted immediately, following the manufacturer’s protocol for the RNeasy kit. Final RNA concentrations were measured with a NanoDrop ND-1000. Total RNA was reverse-transcribed using oligo(dT)_18_ primers (Thermo Scientific), random hexamers primers (Thermo Scientific), and avian myeloblastosis virus (AMV) reverse transcriptase (Promega). cDNA was diluted to 5 ng/µL. Gene expression was assayed by real-time quantitative PCR using Maxima SYBR Green qPCR Master Mix (Thermo Scientific) on an ABI 7900HT real-time PCR system. The SYBR Green qPCR reactions contained 10 µL of 2× SYBR Green Master Mix, 2 µL of diluted cDNA, and 8 pmol of both forward and reverse primers. The reactions were run using the following conditions: 50°C for 2 min and 95°C for 10 min, followed by 40 cycles of 95°C for 5 s and 60°C for 30 s. Relative values for each transcript were normalized to 18S rRNA. Gene primers used are listed in Key Resources. For every qPCR run, three technical replicates per sample were used for each gene.

For HCV detection, we used HCV-specific primers and a fluorescein amidites (FAM)-conjugated TaqMan probe (Applied Biosciences) with PrimeTime Gene Expression Master Mix (IDT) as described (17). Sequences were as follows: 5′-CGGGAGAGCCATAGTGG-3′ (forward), 5′-AGTACCACAAGGCCTTTCG-3′ (reverse), and 5′-CTGCGGAACCGGTGAGTACAC-3′ (probe). For quantification of viral copies, an HCV standard was generated by serial dilution of a described JCV1-mKO2 plasmid (59) and used in every qPCR run.

### Droplet digital (dd) PCR to detect HCV

We used Bio-Rad’s QX100 Droplet Digital PCR System to detect HCV at low levels in the organoids. Organoid RNA was extracted and reverse-transcribed to cDNA as described above. For each sample, 40 ng of cDNA was mixed with 10 µL of 2× ddPCR supermix for probes (no dUTP) (Bio-Rad), 18 pmol of forward and reverse primers for HCV (described above), and 4.5 pmol of the HCV FAM probe (described above). This mixture was dispensed into the sample wells of a D8 droplet generator cartridge (Bio-Rad), and droplets were generated using the QX100 droplet generator according to the manufacturer’s instruction. After droplet generation, the reaction mix was transferred to a ddPCR 96-well PCR plate (Bio-Rad), and the plate was sealed. The plate was run on a thermal cycler with the following conditions: 95°C for 10 min, 45× cycles of 94°C for 30 s and 59.4°C for 1 min, 98°C for 10 min, and 4°C for 30 min. After the protocol was completed, the plate was transferred to a QX100 droplet reader for analysis. For every ddPCR run, two technical replicates per sample were used. A water blank and positive control were included in every run. The positive control was cDNA from Huh 7.5 cells infected at MOI = 0.01 with the JC1-mKO2 HCV strain mentioned above.

To detect the negative strand of HCV, we adapted a qPCR assay for ddPCR (33). In our assay, RNA is extracted from organoids and reverse transcription performed with a tagged forward primer (Tag-RC1) and ThermoScript reverse transcriptase (Thermo Scientific). The Tag-RC1 sequence is 5′-GGCCGTCATGGTGGCGAATAAGCCTAGCCATGGCGTTAGTA-3′. For first-strand synthesis, RNA was mixed with Tag-RC1 primer and dNTP and the whole mix was incubated at 70°C for 8 min then 4°C for 5 min. For reverse transcription, ThermoScript reverse transcriptase, enzyme buffer, dithiothreitol (DTT), and RNase inhibitor were added to the reaction. The total mix was incubated at 60°C for 1 h then heated to 85°C for 5 min to terminate the reaction. To degrade input RNA, RNase H was added to the complete reaction followed by incubation at 37°C for 20 min. After this, cDNA was analyzed by ddPCR as described above but with different primers and probe. We used the described tag-specific forward primer (5′-GGCCGTCATGGTGGCGAATAA-3′) and reverse primer RC21 (5′- CTCCCGGGGCACTCGCAAGC-3′) (33). We designed a HEX-conjugated Taqman probe (Applied Biosciences) compatible with these primers with sequence 5′- AGTGTCGTACAGCCTCCAGGC-3′.

### Whole-mount organoid staining

Organoids were processed for imaging as previously described (23). Briefly, organoids were removed from BME2 with three times cold PBS washes then fixed in 2–3% paraformaldehyde for 30–60 min and washed three times in PBS. Fixed organoid samples were stored at 4°C for up to 2 months.

For staining, organoids were blocked in PBS supplemented with 0.5% Triton X-100, 1% DMSO, 1% bovine serum albumin (BSA), and 1% donkey or goat serum. Organoids were blocked overnight at 4°C. Blocking solution was then removed and replaced with blocking solution containing primary antibodies diluted 1:250. Organoids were incubated with primary antibodies for 48 h at 4°C. After this, organoids were washed three times in PBS and incubated overnight at 4°C with secondary antibodies diluted 1:250 in PBS. Organoids were washed three times in PBS and stained with Hoescht before visualization. Organoids were imaged on a Zeiss Lightsheet Z.1. Images were processed using a combination of the Zeiss software, ImageJ 1.51f, and Imaris 9.3. Primary antibodies we used include CD81 (BD Pharmigen, JS-81), claudin-1 (Thermo Scientific, 2H10D10), HNF4α (Cell Signaling Technology, C11F12), albumin (Sigma-Aldrich, HSA-11), and ZO1 (Thermo Scientific, 1A12).

### RNAScope

For RNAScope, organoids were fixed as described above, except incubation in 2% paraformaldehyde was extended overnight. The RNAScope kit, HCV probe (RNAscope Probe- V-HCV-pool; cat#423291), and 3-plex positive control probe were purchased from ACDBio. To process and stain fixed organoids, we followed the manufacturer’s instructions. Organoids were imaged on a Zeiss Axio Observer Z.1 and Zeiss Lightsheet Z.1. Images were processed using a combination of the Zeiss software, ImageJ 1.51f, and Imaris 9.3.

### HCV infection of Huh 7.5 cells

Huh 7.5 cells were infected with a previously described monocistronic infectious clone of HCV1_JC1_ encoding orange fluorescent protein and a blastocidin-resistance gene (39, 59). Cells were infected at MOIs of 0.01, 0.1, and 0.2. Seven days later, cells were processed for single-cell RNA sequencing as described below.

### HCV infection of organoids

HCV2 EM and DM day 3 organoids were spin-infected with patient-matched virus as follows. Organoids were collected and lightly dissociated by a 3-min incubation at 37°C with TrypLE. Cells were then mixed with sera (HCV titer of 3.5e6 IU/mL) at an MOI of 450 IU/organoid in a 24-well suspension culture plate. The plate was centrifuged at 600 × *g* for 1 h at room temperature, followed by a 2h incubation at 37°C. After this, cells were collected and washed three times in basal media. Washed cells were centrifuged at 250 × *g* at 4°C for 5 min and then seeded in fresh BME2 drops in a new 24-well suspension culture plate. Every 2–3 days, RNA was harvested as described above. On day 9 post-infection, EM organoids were thinly passaged.

### DAAs and IFN-α treatments

Sofosbuvir and velpatasvir were purchased from Selleckchem (cat: S2794 and S3724). Ten millimolar stock solutions of each were made up in DMSO and diluted to the final concentrations used (10 µM for sofosbuvir, 100 nM for velpatasvir). Organoids were dosed two times over 6 days, either in EM media or in DM media. Recombinant human IFN-α was purchased from R&D Systems (cat: 11101-1). It was diluted more than 100-fold to achieve the final concentration of 1,000 U/mL and dosed on the same schedule as the DAAs.

### Single-cell RNA sequencing

Single-cell RNA sequencing was performed using the 10X Genomics Chromium System (26). EM and DM organoids samples were processed within 1 month of starting the organoid culture, or within 3 weeks of thawing a frozen stock. To prepare single-cell suspensions for organoids, samples were incubated with TrypLE for 10–15 min, washed once with basal media, and flowed through 40 µm cell strainers to remove cell aggregates. Cell and viability counts were performed using trypan blue and hemocytometers. Viability was >75% for all organoid samples. To prepare single-cell suspensions for Huh 7.5 cells, samples were incubated with 0.25% Trypsin-EDTA solution (Gibco) for 10–15 min, washed once with DMEM, and flowed through 40 µm cell strainers to remove cell aggregates. Viability was >94% for all Huh 7.5 samples. Single-cell suspensions were concentrated at 12,000 target cells in 30 µl of basal media due to an estimated lower retention rate for hepatocytes per discussion with 10X Genomics.

Single-cell RNA-seq libraries were generated using either the Chromium Single-Cell 3′ Library and Gel Bead V2 Kit or the Chromium Single-Cell 5′ Library and Gel Bead V1 Kit following the manufacturer’s protocols. For the 5′ Library and Gel Bead Kit, we added a custom HCV capture oligo (5′-AAGCAGTGGTATCAACGCAGAGTACTACCTGGTCATRGCYTCCGTG-3′) to the master mix for reverse transcription at a 1:1 equimolar ratio with the Poly-dT RT Primer. We proceeded directly from cDNA Amplification & QC to 5′ Gene Expression (GEX) Library Construction.

Single-cell libraries were loaded on an Illumina HiSeq 4000 System or NovaSeq 6000 System with an S1 flow cell with the following reads: 26 bases Read 1 [cell barcode and unique molecular identifier (UMI), eight bases i7 index 1 (sample index), and 98 bases Read 2 (transcript)].

### Single-cell data analysis

The Cell Ranger pipeline (10X Genomics, Pleasanton, CA, USA) (Version 2.1 and 3.0) was used to perform sample de-multiplexing, barcode processing, and 3´ UMI counting. FASTQ files were aligned to the human reference data set (GRCh38) from 10X Genomics. When applicable, the human reference data set was appended with the HCV genome (GenBank ID AB047639.1 to align libraries from Huh 7.5 cells infected with a JFH1-derived strain and GenBank ID AF009606.1 to align libraries from HCV1 organoid samples of genotype 1).

For each experiment, filtered gene expression matrices from each sample were read into scVI (version 0.5.0) (27) and jointly analyzed using default hyperparameters. Donor annotation was used as batch factor in all donor-derived samples, and library identifier was used as the batch factor in the Huh 7.5 experiment. The 10-dimensional latent representation from scVI was then clustered, visualized, and explored using VISION (version 2.1.0) (28). One low-quality cluster from the analysis of 5′ scRNA-seq presented in Fig. 4, cluster 5, was removed from visualization.

DEGs were determined for clusters using the Wilcoxon rank sum test implemented in Seurat R Package (version 3.1.5) (60) with min.pct = 0.25. Sequencing statistics, including number of cells per sample, reads per cell, and genes per cell are described in Table S1.

### Gene ontology term enrichment (GOTE) analysis

We performed GOTE analysis using Metascape (version 3.5) (38). Express analysis default settings were used.

### Network propagation integrative analysis

We performed network propagation to integrate the single-cell RNA sequencing data with the HCV virus-host protein-protein interactome using the Pathway Commons network (61). Specifically, we used a heat-diffusion kernel analogous to random walk with restart (also known as insulated diffusion and personalized PageRank) which better captures the local topology of the interaction network compared to a general heat diffusion process. The process is captured by the steady-state solution as follows:


(eq. 1) $$P_{SS}=\alpha (I-(1-\alpha )W{)}^{-1}P_{0}$$

where P_SS_ represents the vector of propagated values at steady state, P_0_ is the initial labeling (genes of interest from molecular studies), W is the normalized version of the adjacency matrix of the underlying network (in this implementation W = AD^−1^ , where A is the unnormalized adjacency matrix, and D is the diagonal degree matrix of the network), I is the identity matrix, and α denotes the restart probability (here, α = 0.2), which is the probability of returning to the previously visited node, thus controlling the spread through the network.

We performed two independent propagations, one for the scRNA-seq and one for the HCV interactome. For the scRNA-seq, we compiled all differentially expressed genes (up or down). For the HCV interactome, we used the 134 unique human preys from the published HCV interactome done in Huh 7 cells, based on an HCV MIST score greater than 0.68 and passing the 99th percentile for compPASS scoring (39). To control for nodes with high degree (i.e., many connections), which due to their heightened connectivity are biased to receive higher propagation scores, we conducted a permutation test. Specifically, we simulated random propagations by shuffling the positive scores to random genes, repeating this 20,000 times for both propagation runs. Next, we calculated an empirical *P*-value by calculating the fraction of random propagation runs greater than or equal to the true propagation run for each gene. We integrated the data by extracting the genes with a *P* ≤ 0.03 in both propagation runs.

The network was created by extracting the subnetwork of significant genes from the same Pathway Commons network visualizing interconnected genes using Cytoscape (62). The resulting network was clustered into subnetworks using the GLay Cytoscape plugin (63). One cluster was omitted from visualization in Fig. 6 because it contained less than five genes; information about this cluster is included in Data S3.

## Data Availability

Raw and derived data are deposited on a protected server at Gladstone and can be accessed upon request. The raw genetic sequencing data of patients are not approved for sharing.

## SUPPLEMENTAL MATERIAL

Data S1 mbio.01318-23-s0001.xlsx Differentially expressed genes by cluster from 3' scRNA-seq.

Data S2 mbio.01318-23-s0002.xlsx Differentially expressed genes by cluster from 5' scRNA-seq.

Data S3 mbio.01318-23-s0003.xlsx Network propagation analysis results.

Supplemental Figures mbio.01318-23-s0004.pdf Figures S1 to S6.

Supplemental Legends mbio.01318-23-s0005.docx Legends to supplemental material.

Table S1 mbio.01318-23-s0006.docx Single-cell RNA-sequencing statistics for ;iver organoid samples.

ASM does not own the copyrights to Supplemental Material that may be linked to, or accessed through, an article. The authors have granted ASM a non-exclusive, world-wide license to publish the Supplemental Material files. Please contact the corresponding author directly for reuse.
