# Supplementary material for: Hepatitis C virus infects and perturbs liver stem cells
Source: mBio. 2023 Nov 8;14(6):e01318-23. doi: 10.1128/mbio.01318-23 (PMC10746249; doi:10.1128/mbio.01318-23)

Supplementary Figure 1

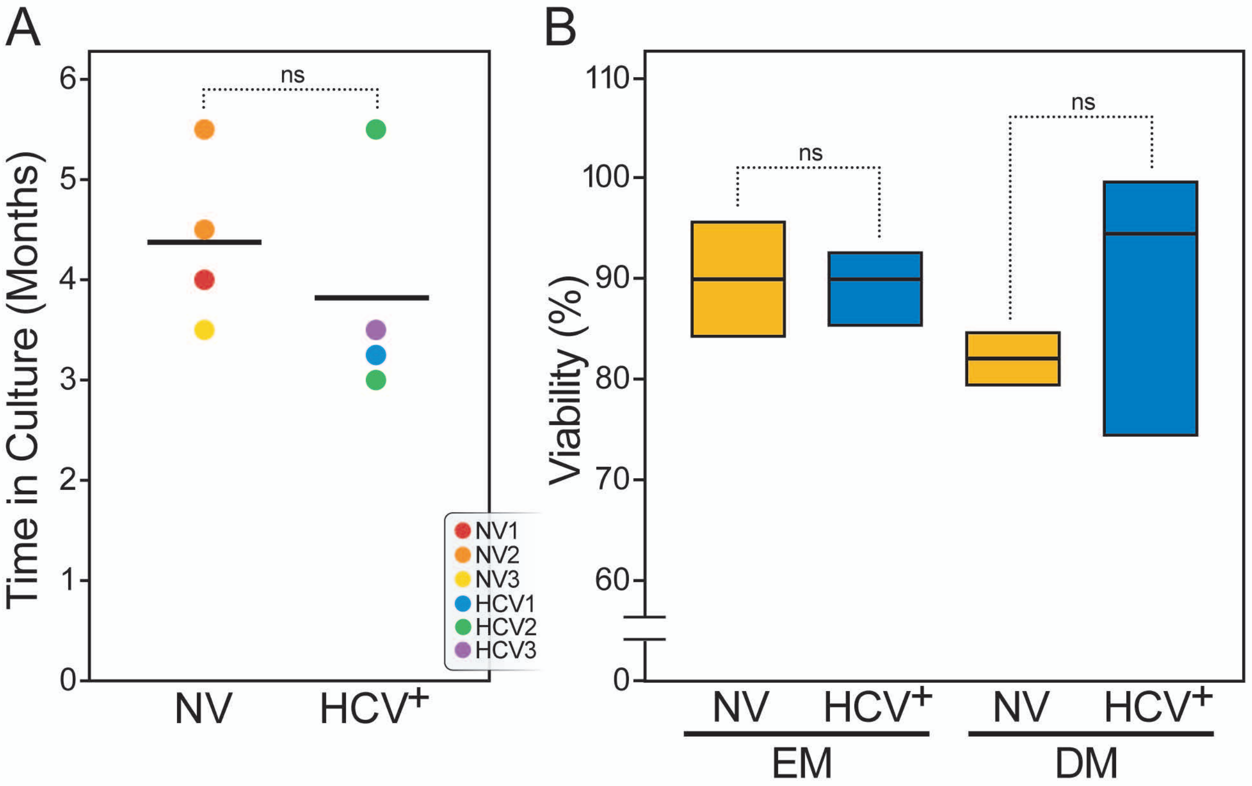

Supplementary Figure 2

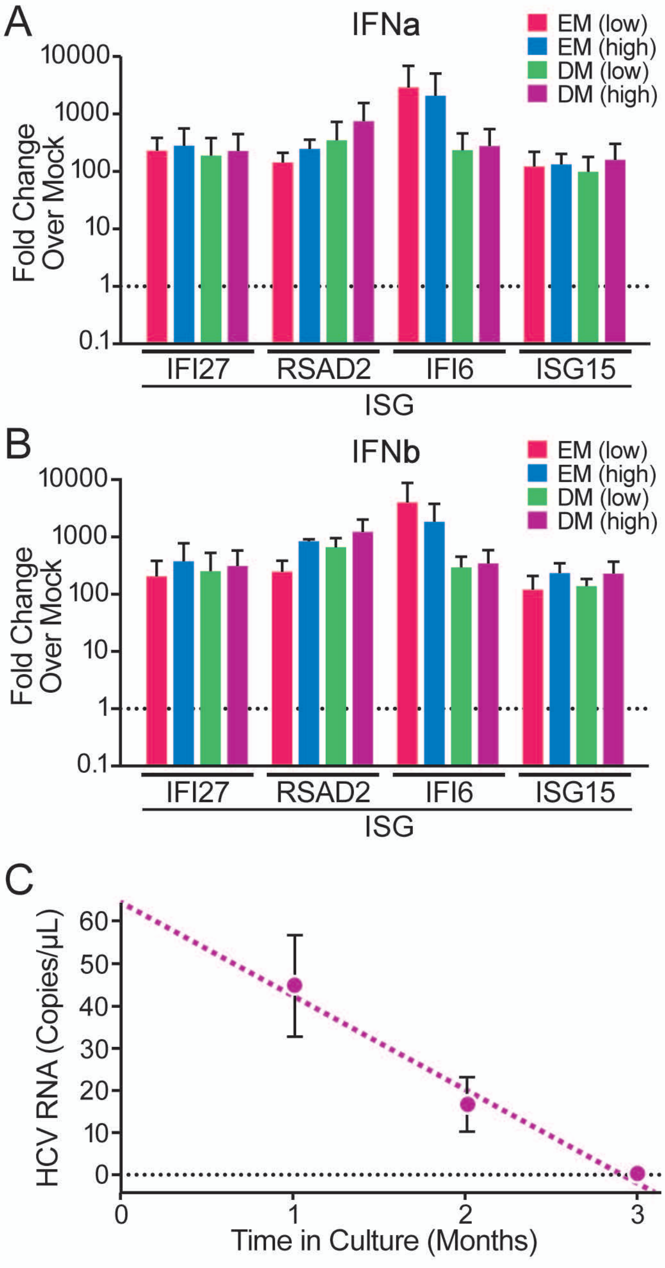

Supplementary Figure 3

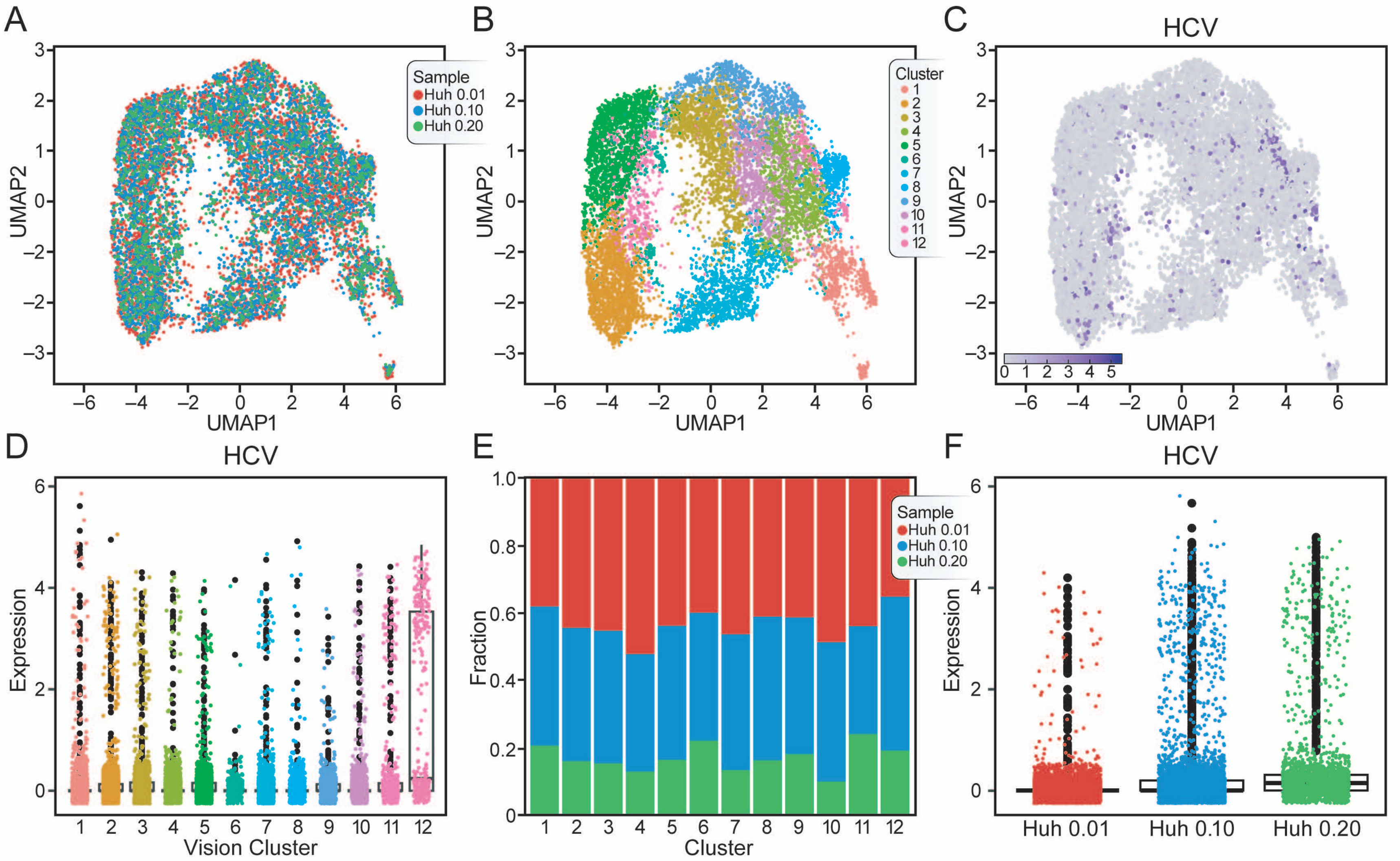

Supplementary Figure 4

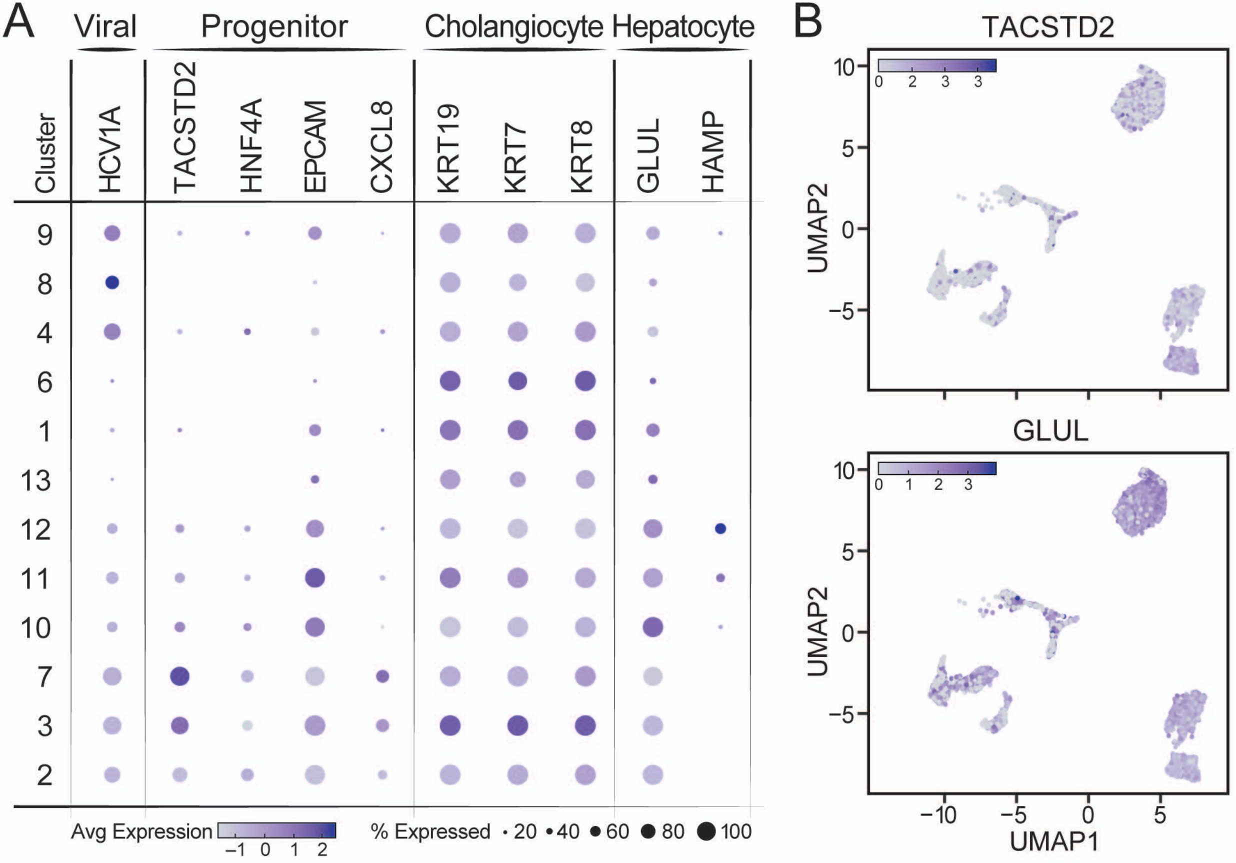

Supplementary Figure 5

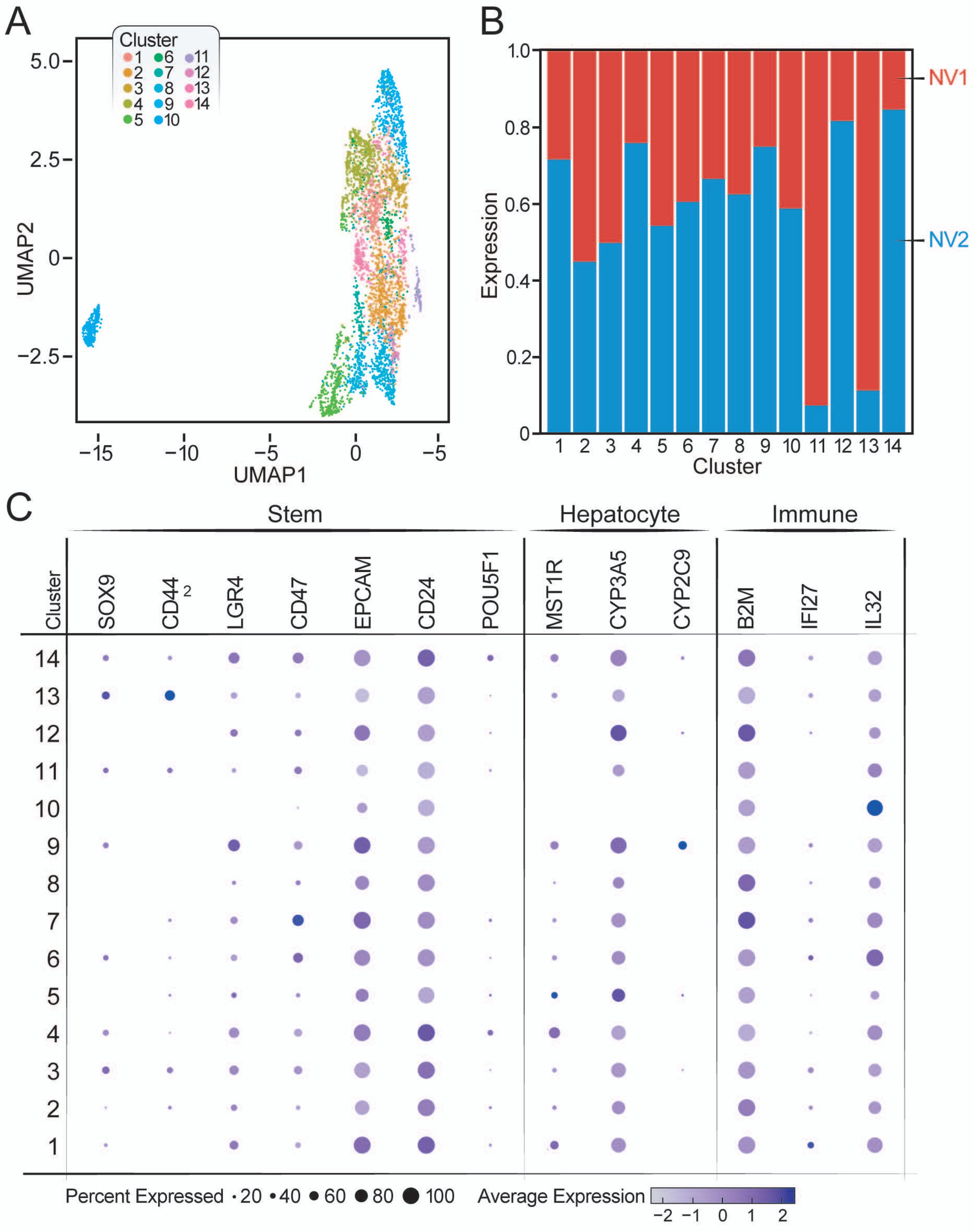

Supplementary Figure 6

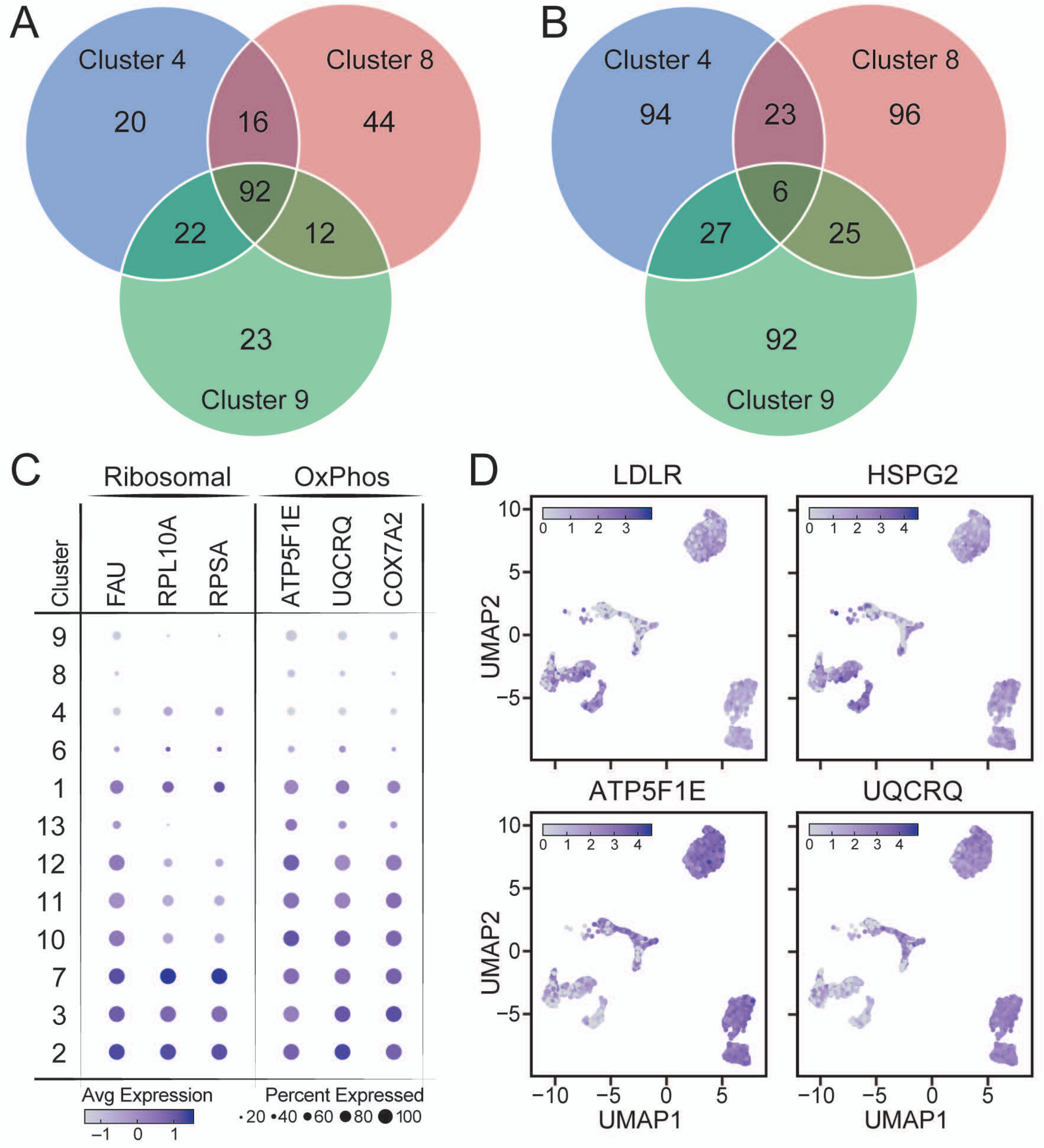

Supplement: Supplemental Figures — Figures S1 to S6. [file mbio.01318-23-s0004.pdf]
