# Supplementary material for: Hepatitis C virus infects and perturbs liver stem cells
Source: mBio. 2023 Nov 8;14(6):e01318-23. doi: 10.1128/mbio.01318-23 (PMC10746249; doi:10.1128/mbio.01318-23)
Supplement: Table S1 — Single-cell RNA-sequencing statistics for ;iver organoid samples. [file mbio.01318-23-s0006.docx]

| Sample Name | Library Kit | Cells | Reads per Cell | Genes per Cell | Median UMI per Cell | Sequencer |
| --- | --- | --- | --- | --- | --- | --- |
| NV1 EM | 3’ | 2,603 | 25,760 | 2,399 | 8,017 | HiSeq 4000 |
| NV2 EM | 3’ | 1,971 | 95,843 | 4,005 | 20,336 | NovaSeq 6000 |
| HCV1 EM | 3’ | 2,702 | 73,456 | 4,435 | 21,758 | HiSeq 4000 |
| HCV2 EM | 3’ | 2,848 | 43,584 | 3,309 | 13,252 | HiSeq 4000 |
| HCV3 EM | 3’ | 2,835 | 41,098 | 2,823 | 9,654 | HiSeq 4000 |
| NV1 DM d10 | 3’ | 1,795 | 34,531 | 2,459 | 7,675 | HiSeq 4000 |
| NV2 DM d10 | 3’ | 2,746 | 70,761 | 3,008 | 9.847 | NovaSeq 6000 |
| HCV1 EM | 5’ | 1,620 | 292,982 | 4,635 | 24,635 | NovaSeq 6000 |
| HCV1 DM d3 | 5’ | 2,898 | 187,140 | 4,282 | 21,792 | NovaSeq 6000 |
| HCV1 DM d6 | 5’ | 5,567 | 92,391 | 2,282 | 19,981 | NovaSeq 6000 |
| Huh 7.5 MOI = 0.01 | 5’ | 5,352 | 278,035 | 6,159 | 38,210 | NovaSeq 6000 |
| Huh 7.5 MOI = 0.1 | 5’ | 4,732 | 264,293 | 6,286 | 42,120 | NovaSeq 6000 |
| Huh 7.5 MOI = 0.2 | 5’ | 1,874 | 758,599 | 6,912 | 51,727 | NovaSeq 6000 |
